# Preparation methods, applications, toxicity and mechanisms of silver nanoparticles as bactericidal agent and superiority of green synthesis method

**DOI:** 10.1016/j.heliyon.2024.e36539

**Published:** 2024-08-21

**Authors:** Godfrey Michael Shayo, Elianaso Elimbinzi, Godlisten N. Shao

**Affiliations:** University of Dar es Salaam, Mkwawa College, Department of Chemistry, P.O. Box 2513, Iringa, Tanzania

**Keywords:** Silver nanoparticles, Toxicity, Biocidal activity, Antibacterial mechanism

## Abstract

Silver nanoparticles (SNPs) are a type of nanomaterial with wide applications in water treatment, medicine, food packaging, and industrial processes. Their unique optical, electrical, thermal conductivity, and biological properties distinguish them from other metal ions and liken them to noble metals like gold and copper. The present review explores the diverse applications, preparation techniques, mechanism of action of SNPs, and properties of SNPs focusing on their bactericidal activities and potential impacts on human health. Different preparation methods, encompassing chemical, physical, and biological techniques, were reviewed and analyzed to comprehend their effect on the properties and applications of SNPs. Studies revealed that the SNPs exhibit excellent antibactericidal properties. Mechanisms underlying their antimicrobial effects were explored, primarily focusing on pathogen-scavenging activities. Despite the promising benefits of SNPs, their potential toxicity to human health must be carefully managed. Regulatory standards, such as those set by WHO and USEPA; establish a maximum tolerable limit of 0.1 mg/L to mitigate health risks associated with SNP exposure. It is recommended to continue research into safer applications and alternative formulations of SNPs to minimize potential health risks while maximizing their beneficial applications across different industries.

## 1 Introduction

Silver nanoparticles (SNPs) are one of the types of zero-dimensional materials with distinct morphologies and sizes between 1 and 100 nm [1]. These nanomaterials can be prepared from silver metals as the prior precursor. However, other materials in the preparation process may be required to accompany the preparation accomplishment. Silver, copper, and gold are considered noble metals on the periodic table. Therefore, metallic nanoparticles made from these noble metals have drawn a lot of attention due to their unique chemical, biological, and physical characteristics as compared to other metals [2,3]. An unconventional antibacterial property of SNPs initially sparked interest in the biomedical evaluation of SNPs in humans’ daily lives [4,5]. This, therefore, draws lots of interest in SNPs' applications in a variety of fields, including health industry [6], cosmetics [7], food storage and packaging [8], textile coatings [9], and some environmental applications such as water disinfection [10].

Experimental studies have examined the antibacterial efficacy of nano-silver-containing biomaterials against a variety of therapeutically significant planktonic and sessile pathogenic microorganisms, including bacteria [11,12]. The remarkable antibacterial activity of SNPs is a great concept to take into consideration when developing and implementing new and improved nano-silver-based biomedical products (anticancer agents, drug delivery platforms, orthopedic materials and devices, bandages, antiseptic sprays, and catheters) [13]. The use of SNPs in biomedicine and environment applications necessitates the ongoing development of affordable SNP production technologies [14]. The development of safe, facile, environmentally benign, and cost-effective preparation methods for SNP is necessary for the formation of final products with appreciable properties for biomedical applications. Additionally, a thorough understanding of the associated physicochemical peculiarities, in vitro and in vivo effects, bio-distribution, safety control mechanisms, pharmacokinetics, and pharmacodynamics of SNPs is paramount [13].

Generally, the catalytic and antibacterial properties of SNP depend on their size, size distribution, structure, shape, and physical-chemical surroundings. Particle size is one of the most crucial parameters as it determines the fundamental properties of the material. For instance in biological systems, size is reported to influence several crucial aspects and processes including drug targeting, delivery, and distribution [10,13]. The smaller the silver nuclei, the more effective the SNPs' antimicrobial activity [15]. Therefore, controlling size and size distribution are crucial attributes of final products with improved performance [15]. Common ways to control SNP's size and size distribution is to vary the reducing agents and stabilizers during preparation.

Meanwhile, the mechanism of SNP antibacterial activity has been widely reported. *E. coli, Vibrio cholerae, Pseudomonas aeruginosa*, and *Salmonella typhi* are the four distinct gram-negative bacteria on which Morones et al. [16] reported the antibacterial activity of SNP. Morones et al. [16] propose that SNPs attach themselves to the cell membrane's surface, infiltrate bacterial cells, and finally release Ag^+^ ions that kill the bacterial cell. This process is thought to disrupt the cell membrane's function. According to Klaivan et al. [17], the binding of an antimicrobial agent to the negatively charged bacterial cell wall destabilizes the cell envelope and changes its permeability. To provide a comprehensive overview of this topic, this review article employed a rigorous research methodology. Key search terms and logical operators were utilized to meticulously sift through academic databases, including Google Scholar, Z-library, ResearchGate, Scholar CiteSeerx, and ScienceDirect. The aim was to capture a broad spectrum of research methods and the latest findings in the field. The process demanded advanced critical thinking to assess and combine information from various sources, such as peer-reviewed studies, patents, and existing reviews, into a unified and informative overview of the subject matter.

The present review discusses various preparation techniques engaged in the synthesis of SNPs such as chemical, physical, and biological (green chemistry) methods. It also discusses the toxicity and poisonous qualities of SNPs concerning human health when the concentration of these nanoparticles surpasses the upper limit that the human body can withstand. The mechanisms by which SNPs function; specifically how they inactivate bacteria are also highlighted. Given the diverse range of techniques for preparing SNPs, each with its strengths and weaknesses, it is essential to critically examine these methods. Chemical methods for instance offer precise control over nanoparticle characteristics but often require toxic reagents and rigorous purification steps. Physical methods are cleaner but usually necessitate high-energy consumption and specialized equipment. In contrast, biological or green synthesis methods, while environmentally friendly, can be inconsistent and difficult to scale up. By comprehensively evaluating these methods, researchers can select the best approach for specific applications, reconcile conflicting research results, and identify opportunities to improve SNP production in terms of efficiency, safety, and sustainability. This review also aims to bridge knowledge gaps and lay the groundwork for future advancements in SNP research and industry.

## 2 Preparation methods of SNPs

The top-down strategy and the bottom-up approach are generally the two most common ways that can be used to prepare nanoparticles. While the mechanical process of ball milling involves controlled erosion of a solid mass, the top-down approach involves milling a solid mass into smaller pieces. This is done to create nanopowders with finer structures or irregular shapes [18]. However, this technique frequently results in contamination from the milling medium or the environment, as well as nanomaterials with relatively large grain sizes ranging from 200 to 300 nm [18]. In the bottom-up method, the nanomaterial is constructed atom by atom over four steps: first, a precursor is condensed to a solid phase, triggering the formation of multiple nuclei; next, growth is achieved on the nuclei; and finally, stabilization brings the process to an end and produces the desired size [19]. Several reported techniques have been used to synthesize metal nanoparticles in recent years. These techniques could be physical [20]; [null]; physico-chemical [21,22]; chemical [[23], [24]]; or biological synthesis methods [15,25]. The inherent flexibility of silver metal and silver-based compounds makes all these methods feasible [25,26].

**Fig. 1:**
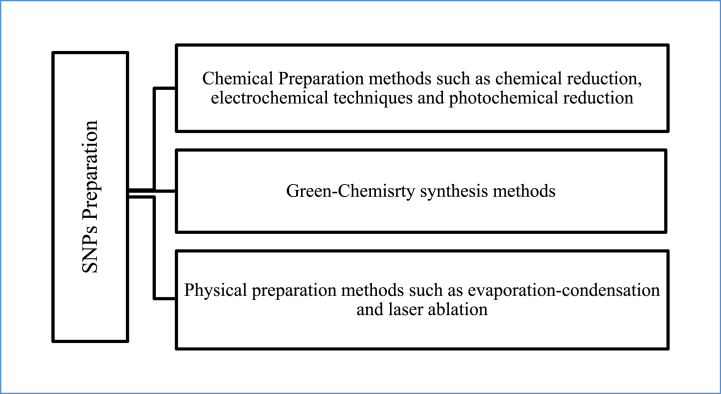
The preparation methods used in SNP synthesis using chemical, green-chemistry, and physical methods. (For interpretation of the references to color in this figure legend, the reader is referred to the Web version of this article.)

The most common methods are chemical ones, including photochemical reduction, electrochemical procedures, and chemical reduction. The other most significant physical preparation techniques are evaporation-condensation and laser ablation. Additionally, biological techniques set the pace due to their obvious environmental benefits. Fig. 1 shows a schematic representation of the three techniques for creating SNPs.

### 2.1 Chemical methods

Chemical methods are the most commonly used approaches for synthesizing SNPs with appealing properties. The chemical synthesis methods involve chemical reduction using organic and inorganic reducing agents [27]. The process seemed simple and secure before the development of alternative methods such as green synthesis. This was due to its minimal costs and ability to produce reliable and reproducible experimental data. It has been reported that utilizing silver nitrate as a salt precursor, ascorbic acid in the aqueous phase can maintain optimum suspensions of zero-valent silver particles (colloidal silver) [28,29]. Nonetheless, Ag^+^ in aqueous or non-aqueous solutions has been reduced using a variety of accessible reducing agents. These consist of N, N-dimethylformamide, poly (ethylene glycol)-block copolymers, polyol process, elemental hydrogen, sodium citrate, sodium borohydride (NaBH_4_), and Tollens' reagent [30].

### 2.2 Physical preparation methods

Physical approaches also create SNPs with small size distribution [31]. However, the drawback of this approach is that it requires enormous consumption of energy. Using the flame aerosol approach, it is possible to create SNPs supported on nanostructured SiO_2_, enabling exact control over the concentration and size of silver (Pratsinis, 2010). Flame spray pyrolysis has been used in forming narrow Ag/silica nanoparticles [32]. Size (surface area), shape, surface charge and coating, aggregation, and dissolution rate are all crucial physicochemical factors to consider when analyzing SNPs, biological interactions, and consequences. A smaller particle's increased surface area gives it a higher toxicity potential [33]. Studies have shown that the surface charges of SNPs' coatings influence their biological effects, which may impact how SNPs interact with living cells of the organism [34]. Compared to SNPs, bulk silver has a larger surface area and less reactivity.

Silver nanoparticles (SNPs) can be produced with high purity without the use of chemical reagents thanks to physical synthesis methods. The physical synthesis method is also one of the commonly employed synthesis techniques over the chemical method. One popular method is laser ablation, which is irradiating a silver target with a pulsed laser in a liquid medium to form SNPs [35]. The size and shape of these nanoparticles can be precisely controlled by adjusting the laser parameters and the properties of the liquid medium [36]. This method is noteworthy for its simplicity and capacity to produce contamination-free nanoparticles, which makes it appropriate for use in biomedicine and electronics [37]. Additionally, it has been shown that laser ablation in liquid produces SNPs with high stability and uniformity [38]. Ball milling is another popular physical synthesis method that uses high-energy ball mills to grind bulk silver into nanoparticles. This is an efficient and scalable technique that produces SNPs with distinct morphological characteristics depending on the milling duration and intensity ([39]; Li et al., 2018). Ball milling is especially beneficial because it can yield significant amounts of nanoparticles with a relatively simple setup. Physical vapor deposition (PVD), in which silver is vaporized in a vacuum chamber and then condensed to form nanoparticles, is another efficient method. PVD offers precise control over nanoparticle size and distribution by manipulating deposition parameters, making it ideal for applications requiring uniform particle coatings [40]. Additionally, the method permits control over particle size distribution by altering the milling parameters [39].

### 2.3 Green synthesis method

Green synthesis of silver nanoparticles (SNPs) has emerged as a sustainable and eco-friendly alternative to conventional chemical and physical methods. This approach leverages biological entities such as plants, bacteria, fungi, and algae to reduce silver ions into nanoparticles, thus eliminating the need for toxic reducing agents and stabilizers. Plant extracts are particularly favored due to their rich content of secondary metabolites, which act as both reducing and capping agents. For instance, the use of plant extracts from *Azadirachta indica* (neem) and *Eucalyptus globulus* has been reported to produce SNPs with controlled size and shape, and enhanced antimicrobial properties as well [11]. In addition promising results have been achieved using *Coriandrum sativum* [15]*; Syzygium jambos* [25]*; Lantana camara* [41]*; Prunus yedoensis* [42]*; Adenium obesum* [43]*; Coffea arabica* [44]*; Bunium persicum* [45]*; Vigna radiate* (Kumar & Jyoti, 2015)*;* and *Microsorum pteropus* [46] as well. Similarly, fungi such as *Aspergillus niger* have been utilized to biosynthesize SNPs signifying the role of fungal proteins in the stabilization process [43,47]. The algal-mediated synthesis also demonstrates a promising route, with algae like *Chlorella vulgaris* facilitating the formation of SNPs under mild conditions [15]. This method not only aligns with green chemistry principles but also offers advantages such as scalability and cost-effectiveness. Furthermore, the biologically synthesized SNPs often exhibit superior biocompatibility and reduced cytotoxicity compared to their chemically synthesized counterparts [25]. These attributes make green synthesis an attractive option for applications in medical and environmental fields.

Many variables, including the kind of biological material, the concentration of silver ions, pH, temperature, and reaction time, affect the synthesis of SNPs utilizing green methods. The size and shape of the generated nanoparticles, for instance, can be greatly influenced by the type of plant extract used. The aqueous extract of Coriandrum sativum (coriander) leaves formed spherical SNPs with an average size of 20–30 nm, as shown by Dutta et al. [25] and Singh et al. [26]. Reaction parameters like pH and temperature can also be quite important. It has been found that lowering the reaction's temperature in neutral pH circumstances promotes the creation of smaller nanoparticles [48]. Additionally, the presence of specific phytochemicals such as flavonoids, terpenoids, and alkaloids in the plant extracts contributes to the reduction and stabilization processes. For instance, flavonoids have been identified as key reducing agents in the synthesis of SNPs using *Camellia sinensis* (green tea) extract [49].

**Fig. 2:**
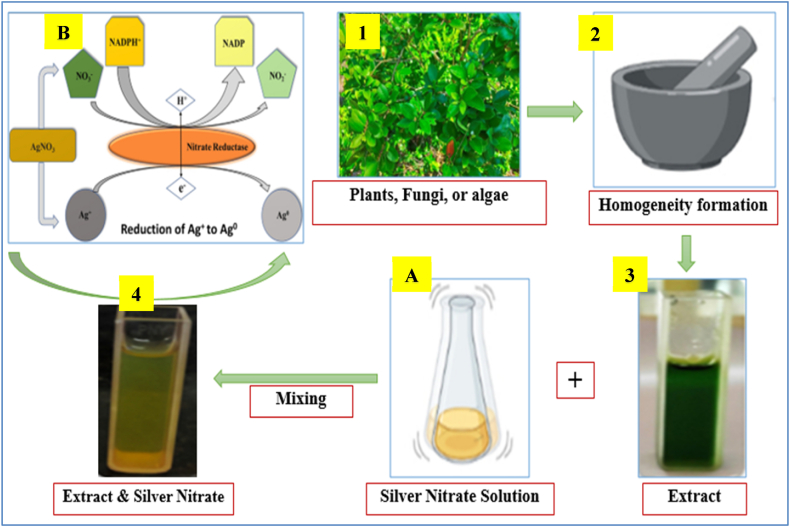
Schematic diagram presenting the preparation of SNPs through the green synthesis method. The image is adopted from Giri et al. (2022) with some modifications. (For interpretation of the references to color in this figure legend, the reader is referred to the Web version of this article.)

The green synthesis method not only reduces the environmental impact but also enhances the biological activity of the nanoparticles. Studies have shown that SNPs synthesized using green methods exhibit potent antibacterial, antifungal, and anticancer activities, making them suitable for a wide range of biomedical applications [49]. The integration of green synthesis techniques in nanotechnology is therefore anticipated to boost sustainable practices in the production of nanomaterials. Fig. 2 illustrates the schematic diagram of the preparation method, highlighting key steps such as the extraction of bioactive compounds from plant materials, followed by their interaction with silver ions. These bioactive substances stabilize the reduced silver ions to form SNPs, preclude agglomeration, and yet lead to the formation of homogeneous particle size.

Fig. 2 indicates that the process follows several steps: (1) Obtaining the plant, algae or fungi extract by collecting fresh leaves, washing them thoroughly, and drying them at room temperature. (2) To make an aqueous extract, grind the dried leaves into a fine powder and combine them with distilled water. (3) Heat the mixture while stirring it constantly to guarantee the consistent extraction of bioactive components. Filter the extract once it has heated up to get rid of any solid residues and leave a clear solution. A) Making a silver nitrate (AgNO_3_) solution at a specified concentration. Next, combine the plant extract in water in a 1:1 ratio, and stir the mixture at room temperature to plate 4. To guarantee total silver ion reduction, let the reaction run for a few hours. (4) Purify the synthesized silver nanoparticles by centrifugation, washing them with distilled water to remove any unreacted plant extract or silver ions, and drying them to obtain the final product as in B.

## 3 Applications of SNPs

Silver is used in clothing, cosmetics, and home appliances. It's worth citing some examples, such as inorganic composites with a delayed silver release rate, which are presently used as preservatives in a variety of cosmetic products [50]. The extraordinarily effective broad-spectrum antibacterial action of SNP products, such as textiles, food storage containers, antiseptic sprays, catheters, and bandages, is one of the primary directions for product development [13]. Another popular use at the moment is silica gel microspheres that have been combined with a silver thiosulfate complex and added to plastics to provide long-lasting antibacterial protection [16]. These substances could be employed in pharmaceuticals and medical equipment since they work well against a variety of bacteria resistant to common antibiotics [51,52]. By denaturing enzymes, silver inactivates bacteria in a variety of ways [53,54]. The ability of SNPs to deactivate microorganisms has been widely investigated. Water-borne microorganisms like *Pseudomonas aeruginosa* and *Aeromonas hydrophila* are normally deactivated by silver. It works well as a secondary disinfectant as well. SNPs' biocidal activity against *Escherichia coli* was studied was examined using scanning electron microscopy and transmission electron microscopy. It was found that *E. coli* cells were injured and developed “pits” in their cell walls upon interaction with silver [55]. To expand their biological uses, SNPs must be synthesized with carefully managed morphological and physicochemical qualities for physiological use in human beings.

Within the range of 10 years, several ideas from different research appliances of SNPs are discussed in Table 1. Table 1 indicates that the presented uses of SNP technology are inevitable because of their availability in the environment. In these reports, the green synthesis method has been widely adopted. This is because green synthesis methods are evolving very rapidly with cost-effective, eco-friendly, and precise properties in their preparations and synthesis processes. It was demonstrated that the biological characteristics of the nanoparticles were significantly impacted by the size of the produced SNPs. It has been stated that the antibacterial capabilities get stronger as the size gets bigger. Research has demonstrated that manufactured SNPs can inactivate a variety of bacterial infections, including *S. aureus* and *E. coli*, depending on how efficient they are. Additionally, these nanoparticles enhance the antimicrobial properties of prepared films, thereby prolonging the lifespan of the products they are applied to.

**Table 1:** The applications, preparation methods, the need for their preparation, Effective sizes, and efficiency of the SNPs on the intended products.

Application	Preparation method	Properties corrected and expected Treatment	Effective size(s) of SNPs prepared	Efficiency of SNPs	References
Water disinfection	In-situ reduction method	Bacterial contaminants from drinking water that is, *E. coli* and *coliform*	The SNP-CF has mesopores with pore volumes of 0.02 cm^3^/g and pore diameters ranging from 2 to 6 nm (with 0.04 ppm of Ag used)	The filter exhibited excellent antibacterial action, eliminating approximately 100 % of *coliform* and *E. coli*	[56]
	Green synthesis (stem extract of *Erythrina abyssinical*	• pH up to 12 % correction • Hardness decreased by 67 % • BOD decreased by 100 % • Nitrates and chlorides (65 %0	The SNP-CF prepared with 0.005 mg/L of silver AgNO_3_	In general, the filter showed a greater efficiency in the correction of the parameters	[57]
Textile Coating	Green synthesis using *Cassia alata* leaf extract as a reducing agent through a simple immersion process	• *Escherichia coli* bacteria • Inhibiting the growth of bacteria in sewerage water	The generation of spherical SNPs in the size range of 20 nm–119 nm	•Nanocomposite cotton fabrics can be taken into consideration for wound dressing and biomedical applications based on the antibacterial activity results •Even after 15 washings, the fabrics still had antibacterial properties	[58]
	Green synthesis with biogenic SNPs made with honeysuckle extract as a natural stabilizer and reductant	The treated silk demonstrated excellent antibacterial activity against *E. Coli* and *S. aureus*, along with some antioxidant activity	More than 80 % of the particles in the SNP size distribution were smaller than 40 nm, which is within the range of 20–90 nm	•The reduction of microbial attack-induced fibre degradation and the improvement of sanitary features •Even after 30 washing cycles, the antioxidant and antibacterial properties remained strong	[59]
Cosmetics products	Physico-chemical reduction method followed with heating was employed with sodium citrate used as a reducing and stabilizing agent	No significant differences were observed for silver penetration when the SNPs were used in the study	The SNPs, which have nominal diameters of 15 nm and 45 nm, respectively, were created	The silver emitted from dermally applied goods containing SNPs (such as cosmetics or wound dressings) can permeate the skin, especially if the skin is injured •Additionally, cytotoxic and genotoxic action of SNP was demonstrated	[7]
	Green synthesis method using plant-derived gallic acid as a reducing and capping agent	The SNPs obtained were employed for the coloration of bleached human hair owing to its local surface plasmonic absorption	The diatomite-supporting SNPs were spherical and cubical in shape with a narrow size distribution (5.89 ± 1.78 nm)	•Hair fibers treated under optimised conditions display good color fastness toward solar radiation •The biocompatibility of the Ag/diatomite composite, SNPs, and the dyebaths was established by in vitro acute dermal and optical toxicity tests	[60]
Medical appliances	One-step biosynthesis method (that is, in *situ reduction* of Sericin from silkworm Cocoon), Sericin served as a reducing agent	The sericin-silver nanoparticle composite required minimum concentrations of 25 mg/L and 100 mg/L to inhibit and eradicate *Staphylococcus aureus*, respectively, based on an antibacterial activity assay	• Most of SNPs were observed in the size range of 4–20 nm • Good crystalline, size distribution and long-term stability at room temperature	•The synthesis of sericin-silver nanoparticle composite was environmentally friendly and energy conservation and the prepared sericin-silver nanoparticle composite exhibited long-term stability, effective antibacterial activity, and acceptable biocompatibility •The biological applications of this brand-new sericin-silver nanoparticle combination, such as an antibacterial agent and wound treatment, have shown remarkable promise	[61][null]
	Green synthesis using *C. roxburghii* leaf aqueous extract and photosynthesis method for SNPs-saturated cotton dress fabrics	The SNPs-infused cotton clothing materials were produced to reduce and completely eradicate the negative effects of chemically produced medications, such as allergies, rashes, itches, and swelling	The SNPs were spherically shaped with a size range of 10–30 nm	•Offers more information on SNPs in wound dressings to hasten the healing of burn wounds and improve human welfare •It is suggested that the cotton materials with SNPs inserted for burn wound care offer promising futures in smart textiles, for both medical and biological applications	[9]
Food storage and packaging	Green synthesis using *Mimusops elengi* fruit extract in the preparation of SNPs	Creating environmentally friendly and economically viable biodegradable packaging materials with the desired qualities for use in food packaging	Prepared a few spherical particles with size ranges from 25 to 45 nm with added SNPs of 0.05 g/100 g	•It also showed desirable mechanical strength for commercial packaging e.g. the prepared film appeared to be a promising protective packaging material that can extend the shelf life of red grapes by 14 days •A unique composite film that can be used in antimicrobial food packaging was introduced in this work	[40][null]
	Chemical reduction method using Silver nitrate, and sodium citrate in the SNPs preparation. In the study, Cellulose nanocrystals were used as carriers and stabilizers of SNPs	Preparing cellulose nanocrystals/SNPs with antibacterial activities against *E. coli* and *S. aureus*	The spherical SNPs with an average diameter size range of 10–20 nm were prepared	•Strawberries were packaged using the produced SNPs/cellulose nanocrystal papers in ambient settings, prolonging the strawberries' shelf life to 7 days •Greater potential for use as environmentally friendly and functional food packaging materials thanks to improved tensile strength and reduced water vapor and air permeability	[62]
	• Green synthesis using chitosan as a reducing agent • The film composite materials were prepared by a casting-solvent evaporation technique	Preparing chitosan-based films immobilized with SNPs for keeping litchis fresh	The film composite formed stable black SNP spots suggesting that the nano-platelets spontaneously assembled to the sandwich structure	•The produced films demonstrated excellent antibacterial activity, very low cell toxicity, and successfully prolonged the storage life of litchi as a package •Chitosan-based films' •Mechanical, water-soluble, and oxygen transfer rates are all clearly improved by the synergistic impacts of laponite and SNPs	[63]

New kinds of bactericidal materials might be produced using relatively, inexpensive non-toxic nanoparticles. In *E. coli*, the antibacterial effects of SNPs created by a special electrochemical technique were investigated. Another study examined *Staphylococcus aureus, Staphylococcus coli, Aspergillus niger,* and *Penicillium phoeniceum*. Additionally, SNPs have potent bacteria-killing and antifungal activities [64,65]. We assessed the antibacterial activity of SNPs and AgNO_3_ against *E. coli* in terms of growth rate, time dependence, and zone of inhibition. SNPs created a zone of inhibition that measured 1.7 cm compared to 0.8 cm for AgNO_3_, proving that they are potent antibacterial contenders [66]. Another investigation into SNP bactericidal activity against gram-negative bacteria revealed that the effect is size-dependent. The most efficient range of nanoparticle size that allows for direct interaction with bacteria has a diameter of about 1–10 nm within the measured range of 1–100 nm.

For centuries, water and medical equipment have been cleaned using silver ions and silver compounds. The idea of SNPs acting as biocides, such as against *E. coli*, has attracted a lot of interest due to the usage of Ag metals and silver compounds as antimicrobial agents. Additionally, cellulose acetate fibers containing embedded SNPs work well as biocides against *S. aureus* and other gram-positive and gram-negative bacteria. *E. coli, Klebsiella pneumoniae, Pseudomonas aeruginosa,* and *E. aureus* [67]. SNPs can directly harm bacterial cell membranes. Increasing membrane permeability, which can result in a loss of proton motive force, cell de-energization, phosphate efflux, cellular content leakage, and disruption of DNA replication, is the general mechanism by which the release of silver ions from SNPs causes bactericidal activity [68]. Additionally, SNP-alginate composite beads for point-of-use drinking water disinfection have been developed and shown to have appropriate bactericidal efficiency and effective disinfection performance.

A variety of physical, chemical, thermal, electrical, and optical properties are required for the development or synthesis of metal-derived nanomaterials for biomedical applications. While some qualities are more relevant to industrial and environmental applications, others are more relevant to medicinal applications. Research reports that nanoparticles exhibit distinctive and substantially more effective physico-chemical characteristics than their macro-equivalents, which makes them suited to their intended usage in better healthcare [69]. Currently, the universe needs an innovative, strong antibacterial medicine for medical uses since more and more microbiological species develop antibiotic resistance. Due to their unique physical and chemical characteristics, nanoscale materials have become desirable choices. Inhibiting bacterial growth has been achieved by using silver (Ag) salt antibacterial properties in a variety of equipment and applications, including catheters, dentistry, burn wounds, coated medical devices, and water filtration. Furthermore, according to Pal et al. [70], one benefit of using nanoscale SNPs against microbes like *Escherichia coli* and Enterococcus faecium is that they are less likely to develop resistance because of their broad spectrum of activity, in contrast to the narrow targets of conventional antibiotics, which are developing resistance [12]. This capability includes the ability to electrostatically attach to the cell membrane and enter bacteria, where they interact with phosphorus and sulfur-containing materials, such as DNA [71,72]. Furthermore, it has been proven that they impact bacterial cells' respiratory and reproductive chains, which causes cell death [72].

Considering Table 2, the prepared SNPs following different synthesis processes and preparation methodologies show excellent nanoparticles in terms of their application. It is observed that chemical and green synthesis preparation methods dominate preparation methods, while physical methods are expensive and energy-consuming. Preparation techniques are currently ignored. However, green synthesis is picking up pace compared to chemical techniques due to improved factors. The results presented in each study show effectiveness in the analysis of prepared samples. This is particularly the success in improving size and the achievement in the preparation of the samples' antibacterial properties.

**Table 2:** Overview of synthesis processes, preparation methods, and applications of prepared silver nanoparticles (SNPs).

Synthesis Process	Preparation Method	Application(s) of prepared SNPs	References
SNPs were synthesized using Polydopamine used both as reducing and adhesive agents	Two-step chemical process (Chemical reduction method)	• Catalytic application/activity of the composite (that is, SNPs/polyacrylonitrile fiber paper Polydopamine functional) • The synthesized product has good catalytic activity, reducing 4-nitrophenol to 4-aminophenol completely in 30 min	[73]
SNPs were synthesized using *Coriandrum sativum*	Green synthesis method	• The SNPs were synthesized for different applications such as water disinfection and catalytic activities	[15]
SNPs were synthesized using tannic acid-coated viscous textile and further hydrophobic treatment	In-situ growing of SNPs on tannic acid-coated viscous textile	• In textile production industries that is, textiles with durable hydrophobicity and anti-bacterial properties • The prepared textiles showed high static water contact angles (>150°) and excellent antibacterial properties against *E. coli* and *S. aureus* but also retained their excellent antibacterial and superhydrophobicity after 50 washing cycles	[74]
SNPs were synthesized using activated carbon and thermal treatment	In-situ reduction method	• Water disinfection applications • Over approximately 10 weeks, 100 % of *E. coli* and *coliform* were removed via filtering, demonstrating higher antibacterial action in the SNPs/CF preparation	[56]
SNPs were synthesized using *Puranta erecta* leaf extract as a reducing agent	Green synthesis method that is, the Facile biological route	• To be applied in wastewater and drinking water purification. • The prepared SNPs/clay reduced the pH of raw water by 12 %, hardness by 67 %, and 100 % decrease in BOD	[57]
SNPs were synthesized using *Microsorum pteropus* methanol extract	Green chemistry method	• Medicinal applications such as the synthesis of Ag-NPs for drug production due to their antioxidant properties • The antioxidant properties of the Ag-NPs showed 1, 1-Diphenyl-2-picrylhydrzyl reduction and H_2_O_2_ scavenging activities	[46]
SNPs were synthesized using ascorbic acid as both reducing and capping agent	In-situ reduction of metal precursor and graphene oxide using L-ascorbic acid as a green reductant	• Production of antimicrobial nanohybrids alternative drugs due to their synergic properties such that, nanofilters in polymer nanocomposites as cancer therapeutic agent • The prepared nanohybrids showed antibacterial activity against gram-negative and gram-positive bacteria depending on concentration and time	[75]
SNPs were synthesized using a hydroxyethyl cellulose solid-state reduction process	Green chemistry Method (Solid-state redox route	• Production of SNPs for their antibacterial properties to be used in sensor hydrogel • The composite hydrogels are applied to the field of antibacterial strain sensor and the highest gauge factor reached to 4.07	[76]
SNPs were synthesized using *Beaveria bassina* and *Metarhizium anisopliae*	Biosynthesis method that is, the green chemistry method	• SNPs for antibacterial and antifungal properties with potential for various applications • Fungal sporulation was more inhibited by SNPs prepared than vegetative growth but also had good compatibility with fungi	[77]
SNPs were synthesized using ethylene glycol in the presence of polyvinylpyrrolidone	Polyol Method (Reduction method)	• Synthesis of SNP ink, such as conductive ink, which may be employed in a variety of applications, including reference electrode manufacturing • With an average ohmic resistance of 1.53 Ω, the developed SNP ink shows outstanding conductivity	[78]

## 4 Working mechanism of SNPs

Numerous potential pathways of bactericidal activity have been proposed. When SNPs and bacterial membrane components interact leads to structural alterations and subsequent damage, which led to bacterial cell destruction [79]. As an alternative, SNPs stop bacteria's action of the respiratory enzymes and promote reactive oxygen species (ROS) which harm cells. Additionally, the toxicity mechanisms are likely brought on by nanoparticle chemical changes during intracellular operations. Although the exact mechanism of SNPs' antimicrobial action remains unclear, it is theorized that these nanoparticles operate through two primary pathways. They may damage microbial membranes by attaching to cell surfaces, leading to structural changes such as gap formation, destabilization, and leakage of cytoplasm. It has been further reported that the SNPs could cause sub-cellular damage by releasing Ag + ions, which in turn generate ROS or deactivate essential macromolecules like proteins, enzymes, and nucleotides. [[80], [81], [82]]. However, adhesion to microbial cells, production of ROS and free radicals, microbial wall piercing and penetration into cells, and regulation and modulation of microbial signal-transduction pathways are the most important mechanistic modes of SNP-based antimicrobial activities [79]. The presence of proteins, the actions of phosphate and chloride, and other biological components make it easy to isolate metallic silver ions, despite their strong antibacterial properties. Many physicochemical parameters, such as form, size, oxidation and dissolving states, surface charge, and surface coating, have a significant impact on the inherent biocide or biostatic activity of SNPs [79].

**Fig. 3:**
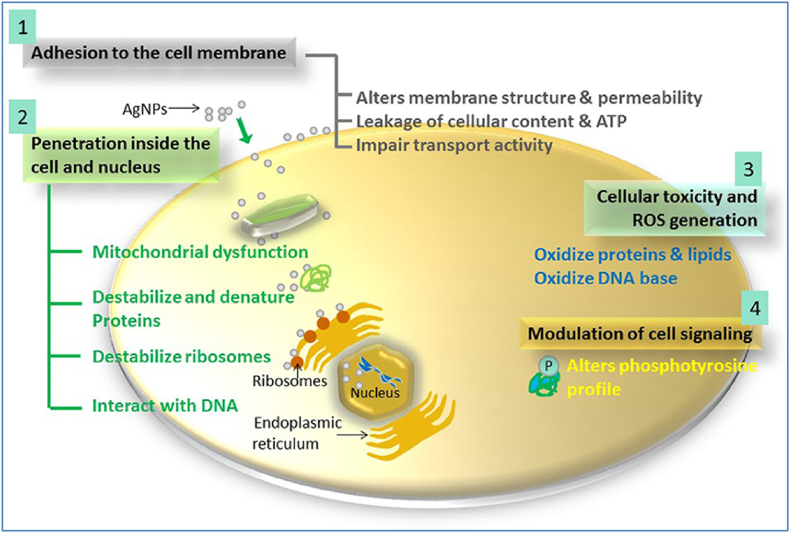
The diagram illustrates a simplified model of how SNP variations affect genes and bacteria.

In the other study, it is reported that SNPs' bactericidal activity is attributed to four distinct mechanistic pathways namely; adhesion to cell walls and membranes; cell penetration and damage to intracellular organelles like mitochondria, vacuoles, and ribosomes; SNP-induced cellular toxicity and oxidative stress brought on by the production of ROS and free radicals; and modulation of signal transducers. In addition to these four well-known processes, SNPs can influence human cells' immune systems by coordinating inflammatory responses, which further suppress bacteria [83]. However, according to Durán et al. [81], only two basic pathways are taken; the nanoparticles likely stick to the bacteria's surface and change the membrane characteristics; and DNA damage may be brought on by SNPs inside the bacterial cell such that, when SNPs dissolve, antimicrobial silver ions are released. The SNPs can interact with thiol-containing proteins in the cell wall and alter their activities, which is regarded as a crucial mechanism of SNPs' antibacterial action [81]. According to Park et al. [72], SNPs have been demonstrated to interfere with DNA replication, affect cell membrane permeability, and produce ROS through proxies [71]. The mechanism of how SNPs work on disruption of the bacterial cell is provided in Fig. 3.

The Fig. 3 illustrates how SNPs (silver nanoparticles) operate against bacteria and other microorganisms through four main steps: 1) Cell Wall and Cytoplasmic Membrane Disruption: Silver ions (Ag^+^) released by silver nanoparticles attach to or penetrate the cell wall and cytoplasmic membrane. 2) Intracellular Effects: Inside the cell, silver ions denature ribosomes and inhibit protein synthesis. 3) Cellular Toxicity and ROS Generation: Silver nanoparticles cause cellular toxicity in bacteria primarily by generating ROS. These ROS damage proteins, lipids, and DNA, leading to cell death. The broken electron transport chain also produces ROS, which disrupts the membrane. 4) Membrane Denaturation and Perforation: Silver nanoparticles accumulate in cell wall pits, causing membrane denaturation. They can also directly traverse the cytoplasmic membrane, potentially releasing organelles from the cell. The image is adopted from Ref. [69].

## 5 Toxicity of silver nanoparticles

To date, there are various studies investigated the potential toxicity of SNPs issue and found that a variety of consumer products release high quantities of SNPs into the environment [84]. For example, washing machines emit SNPs into the environment at an average concentration of 11 g/L [85,86]. In another scenario, SNPs were shown to be released into the environment from outside facades during early runoff episodes in another experiment, with a maximum concentration of 145 g/L [86,87]. The aforementioned instances generate potential environmental and health alarms since the toxicity threat of SNPs may be seen close to consumers, especially in the freshwater ecosystem [1]. The toxicity of SNP may have effects on the human body when in contact with human organs, tissues, and other physiological components. According to Hyun et al. [88], the inhalation toxicity of SNPs had an effect on neutral mucins in the respiratory mucosa of Sprague-Dawley rats exposed to SNPs at doses of 0.5–61 g/m3, but this did not have any toxicity repercussions. Despite the fact that numerous toxicological studies using SNPs have been published, the report asserts that it is still difficult to draw firm conclusions about their toxicity. Because of their varied synthesis methods, sizes, the presence or lack of capping agents, different species, and/or cultivated cells, SNPs may display unique toxicological characteristics ([89]; Gupta & Xie, 2018).

Silver and SNPs may damage the body's reproductive system because they are present in numerous commercial products, including contraceptive devices, food packaging, and feminine hygiene products. Since these products are widely used in various cosmetics and fabrics, a person's skin exposure to toxic SNPs may increase through consumer consumption [90,91]. Depending on the amount of silver coating, the fabric's quality, pH, and sweat production, SNPs can be released from consumer goods. In a different study that used an in vitro methodology, SNPs caused cell death and oxidative stress in human fibrosarcoma and skin cancer cells. Additionally, extensive research has demonstrated that SNPs can enhance cytotoxicity and oxidative stress in human hepatoma HepG2 cells, decrease cell proliferation and chemotaxis in human mesenchymal stem cells, and have a number of other negative impacts [49]. The sizes and dosages of SNPs have a significant impact on the aforementioned toxicities.

Recent studies have highlighted the potential toxicity of SNPs on various biological systems. For instance, research has shown that SNPs can induce cytotoxicity in immune cells, potentially impairing the body's immune response. When it comes to reproductive health, SNPs have been found to adversely affect reproductive organs, causing cellular damage and oxidative stress in ovarian and testicular cells [92]. Additionally, SNP exposure has been linked to cardiovascular issues, with evidence suggesting that they can induce inflammation and oxidative stress in cardiac tissues [93]. The nervous system is also at risk, as SNPs have been shown to cross the blood-brain barrier, potentially leading to neurotoxicity and cognitive impairments [94]. The digestive system is not spared either, with SNPs causing disruptions in gut microbiota and impairing intestinal cell function [95].

Despite these concerns, some studies indicate that SNPs can be non-toxic under certain conditions. For example, the concentration and size of SNPs play a crucial role in determining their toxicity. At lower concentrations, SNPs have been found to be relatively safe for human cell lines, exhibiting minimal cytotoxic effects [96]. Moreover, surface modifications of SNPs can significantly reduce their toxicity, enhancing their biocompatibility and reducing adverse effects on normal human cells [97]. Specific coatings, such as polyethylene glycol (PEG), have been shown to mitigate the toxic effects of SNPs, making them safer for medical and industrial applications [98]. Furthermore, some studies suggest that SNPs might even possess beneficial properties, such as anti-inflammatory and wound-healing capabilities, under controlled usage (Janjua et al., 2015).

Balancing the beneficial applications of SNPs with their potential health risks remains a significant challenge. While their antimicrobial properties are invaluable in medical and industrial settings, the potential toxicity to human health cannot be overlooked. Therefore, rigorous assessment and regulation of SNP usage are essential to ensure their safe application. Future research should focus on understanding the precise mechanisms of SNP toxicity and identifying safe dosage thresholds to mitigate health risks. Additionally, developing advanced synthesis methods to produce biocompatible SNPs can enhance their safety profile. By integrating safety assessments into the development and application processes, the full potential of SNPs can be harnessed while minimizing health risks [99]. Overall, continued exploration of SNPs' toxicological effects and safe usage practices is crucial for their sustainable integration into various sectors.

## 6 Future prospect

Globally, the requirement for nanostructured materials is tremendously increasing due to the possibility of producing convenient materials with improved performance and quality. SNPs are leading the way in nanotechnology due to their unique characteristics. Their adaptability makes them invaluable in various fields. From healthcare, where they can refine medical tools and drug administration, to environmental solutions like water purification, and even electronics, nanoparticles are revolutionizing technology. The future of SNP research and development will likely focus on optimizing their preparation techniques, enhancing their bactericidal efficacy, and ensuring their safe use in mitigating their potential health risks. As understanding of the mechanisms underlying their antimicrobial activities deepens, novel applications in pathogen control and environmental sanitation may emerge. Researchers and innovators are striving to design distinct preparation methods to enhance large-scale production and commercialization of SNPs for myriad applications, particularly for water disinfection activities. Technological developments in eco-friendly and biocompatible synthesis techniques have the potential to improve the safety profile of SNPs and bring them into compliance with regulations that safeguard human health. There are many promising novel approaches to strengthen the application of the SNPs through fabrication of smart materials and medical devices. Sustaining multidisciplinary research will be essential to addressing obstacles concerning environmental effects and toxicity. The use of green chemistry methods not only provides a broad benchmark for SNP preparation but also ensures the availability of precursors and simplicity in the synthesis process [86]. By striking the balance between efficacy and safety, SNPs can play a transformative role in advancing public health, sustainability, and technological innovation.

## 7 Conclusions

The present review demonstrated that SNPs have potential applications in distinct areas. Their potent bactericidal capabilities through their interactions with microorganisms, particularly bacteria have been widely reported. SNPs are very effective in deactivating all forms of bacteria that pose threats to human and animal health. The size of SNPs plays a crucial role in their effectiveness, with smaller particles exhibiting higher antibacterial activity compared to larger ones. This size-dependent efficacy is crucial in optimizing their use in various applications. However, the potential health risks associated with SNPs cannot be overlooked. The toxicity of SNPs necessitates careful management and adherence to regulatory standards, such as the 0.1 mg/L limit set by WHO and USEPA in 1996. It is crucial to strike a balance between the benefits of using SNPs in industries like food packaging, medicine, and water treatment, and the necessity to safeguard public health and safety. It is anticipated that more research on SNP will be carried out to optimize the preparation techniques and enhance their performance while ensuring their safety to mitigate potential health risks and the environment at large.

## Ethics approval and consent to participate

Not applicable.

## Funding

The authors declare that no funds, grants, or other support were received during the preparation of this manuscript.

## CRediT authorship contribution statement

**Godfrey Michael Shayo:** Writing – review & editing, Writing – original draft, Visualization, Conceptualization. **Elianaso Elimbinzi:** Writing – review & editing, Conceptualization. **Godlisten N. Shao:** Writing – review & editing, Supervision, Conceptualization.

## Declaration of competing interest

The authors declare that they have no known competing financial interests or personal relationships that could have appeared to influence the work reported in this paper.

## List of Abbreviations

SNPs: Silver Nanoparticles
WHO: World Health Organization
USEPA: United States Environmental Protection Agency
*E. coli*: *Escherichia coli*
NPs: Nanoparticles
ROS: Reactive Oxygen Species
XRD: X-Ray Diffraction
TEM: Transmission Electron Microscope
SEM: Scanning Electron Microscope
FTIR: Fourier Transform Infrared
UV–Vis: Ultra Violet-Visible
XPS: X-Ray Photoelectron Spectroscopy
EDS: Energy Dispersive Spectroscopy
